# Graphene Oxide (GO) Coating on Reticulated Open-Cell Mullite (ROM) Foams for Enhancing Antibacterial Activity

**DOI:** 10.1021/acsomega.5c04259

**Published:** 2025-11-04

**Authors:** Wadwan Singhapong, Angkhana Jaroenworaluck, Watchara Chokevivat, Pongthorn Suksanong

**Affiliations:** National Metal and Materials Technology Center (MTEC), 61191 National Science and Technology Development Agency (NSTDA), 111 Thailand Science Park, Phahonyothin Road, Khlong Nueng, Khlong Luang, Pathum Thani 12120, Thailand

## Abstract

Graphene oxide (GO) was synthesized by a modified Hummers’ method to study its antibacterial activity in two forms: powders and coating agents. Reticulated open-cell mullite (ROM) foams, fabricated by a replica method using polymer foam as the pore template, were enhanced in their antibacterial activity by coating them with the synthesized GO dispersed in aqueous solutions. To fabricate the mullite foams, nano silica (nSiO_2_), derived from rice husk (RH), was mixed with commercial alumina (Al_2_O_3_) and sintered at 1500 °C for 4 h. The sintered foams were pretreated with HCl acid and dip-coated in GO aqueous solutions prepared with a fixed ratio of GO and deionized water. The dip-coating process was performed 3, 5, and 10 cycles to obtain GO multilayers on the surface of the mullite foams. X-ray computed tomography (XCT) analysis was used to investigate the external and internal microstructures of GO-coated foams and to determine GO layer thicknesses. Antibacterial activities against Gram-negative bacteria (*Escherichia coli*) and Gram-positive bacteria (*Staphylococcus aureus*) by the synthesized GO powders and GO-coated foams were determined based on the standard test ASTM E2149. The synthesized GO powders showed effective antibacterial properties. Microstructural analysis of the GO-coated foams confirmed the successful coating process due to the presence of multilayered GO on the foam surfaces. Ten cycles of GO coating on the foams showed the most effective antibacterial activities against both *Escherichia coli* and *Staphylococcus aureus*.

## Graphical abstract

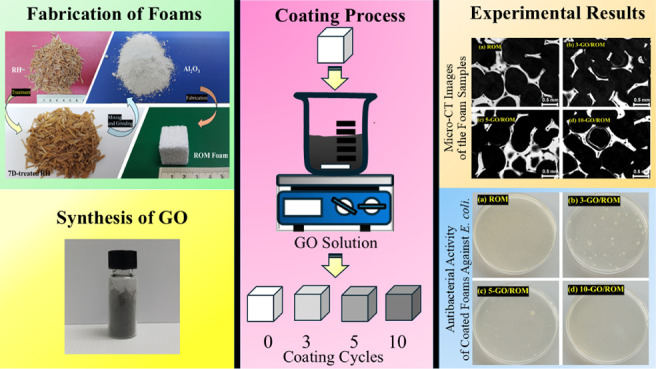

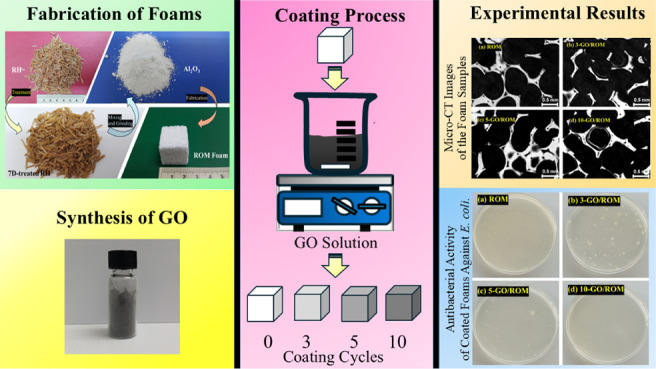

## 1 Introduction

Materials possessing antibacterial properties have attracted great interest due to the personal and global healthcare needs for infection resistance.^1^ It has been known for decades that silver (Ag) and copper (Cu) elements, including their forms as oxides and composites, have been commonly recognized and used as antimicrobial agents in many applications.^2,3^ Recently, photocatalyst materials have also been claimed to be effective antibacterial materials when they are integrated with light illumination. As potential photocatalyst materials, TiO_2_, ZnO and their composites have been recognized and used in various areas because of their effective properties including low costs and stability. Previous studies have reported the antibacterial activities of TiO_2_ nanoparticles under light illumination.^4^ ZnO nanoparticles could be used against microbes with and without the illumination of light.^5–8^ However, as an alternative, researching and finding new types of photocatalyst materials possessing effective antibacterial properties without using any light illumination, or being able to function in low light-intensity areas such as indoor environments, have attracted much attention for the degradation of volatile organic compounds (VOC) and antimicrobial activity.

Graphene (GR), an advanced carbon material belonging to the microstructure of two-dimensional (2D) carbon atoms arranged through sp^2^ hybridization bonding,^9–11^ has been applied in various applications due to its superior thermal, mechanical, optical, and electrical properties.^12^ Recently, GR has been classified as one of the materials possessing antibacterial properties.^1,13,14^ Graphene oxide (GO), one of the GR derivatives, is produced by strongly oxidizing graphene,^15^ which impacts the separation of graphene sheets.^16^ Consequently, GO contains functional groups that give it unique properties, i.e., hydrophilicity, biocompatibility, and antibacterial properties.^9,10,17–20^ Large-scale production of GO, characterized by lower toxicity, ease, and cost-effectiveness, is possible by using the Hummers’ method and its modified Hummers’ method, which are top-down synthesis approaches.^10,18,21,22^

Due to the functional groups on its surface, GO has recently been applied in various kinds of applications, i.e. (i) as an effective adsorbent for water and wastewater treatment^22–26^ and air purification,^27^ (ii) as an electrode material for supercapacitors (SC)^22,28^ and (iii) as an antibacterial agent and biocompatible material for biomedicines.^9,29–31^ Since it is easy to synthesize and use, GO has commonly been used in powder form, which poses a restriction on studies and applications in some cases. Consequently, a few studies have reported methods to produce porous GO in monolithic porous materials by using the freeze-drying method for drying the prepared hydrogel or aerogel.^32^ However, GO coatings or its composites on other materials’ surfaces are considered a proper alternative method to increase its functionalities^33,34^ and to protect corrosive materials, typically metals.^35^

Porous materials have been well-known and used in various industries in practical, relying mainly on the design of pore structures, sizes/distributions, and surface characteristics, formed by using different synthesis or fabrication methods.^36^ It is well-known that pore sizes of materials have been classified into three kinds and defined by the International Union of Pure and Applied Chemistry (IUPAC): (i) micropores (<50 nm), (ii) mesopores (2–50 nm), and (iii) macropores (>50 nm).^36–38^ To address the disadvantages of other porous materials, porous ceramics are generally used in environments requiring chemical corrosion resistance , heat/wear resistance, high hardness, etc.^37,38^ To form the final stage of porous ceramic monolithic foams, the use of pore forming agents in the ceramic process, such as binder agents, before bonding the ceramic particles together during the sintering process at high temperatures, is required to increase the mechanical properties of the end products.^37,38^ The sintering process performed at high temperatures is a crucial parameter that impacts the chemical inertness of the surfaces of the sintered ceramics. To extend the applications of porous ceramics by increasing the functionalities of porous materials, using a second material with specific functional properties to coat the inactive surfaces of the porous ceramics is an alternative and beneficial method.^3^

In this study, open cellular mullite foams were fabricated by using nano silica (nSiO_2_) derived from rice husk as the starting raw material, mixed with commercial alumina (Al_2_O_3_). The surfaces of the sintered foams were further decorated with synthesized GO to form composites for bacterial inactivation of Gram-negative (*Escherichia coli*) and Gram-positive (*Staphylococcus aureus*), compared with the synthesized GO powder and the GO-uncoated foams. The GO layers coated onto the foam were varied by the dipping coating method prepared in this study to evaluate the effectiveness of the layers in promoting antibacterial activity.

## 2 Materials and Methods

### 2.1 Chemicals and Materials

H_2_SO_4_ (98% w/w, Carlo Erba Co., Ltd., Italy), H_3_PO_4_ (85% w/w, RCI Labscan Co., Ltd., Thailand), HCl (37% w/w, Thermo Fisher Co., Ltd., Australia), KMnO_4_ (Thermo Fisher Co., Ltd., Australia), and H_2_O_2_ (>30% w/v, Fisher Scientific Co., Ltd., UK), were purchased via representative companies in Thailand. All chemicals used in this study for the GO synthesis are of analytical grade. For raw materials, alumina powder (Al_2_O_3_) was donated by Rio Tinto Alcan Inc., Canada, and rice husk (RH) was received from a rice mill company in Saraburi province, Thailand. In this study, RH was used as a source of silica to replace commercially available silica.

The Gram-negative (*Escherichia coli*: ATCC 25922) and the Gram-positive (*Staphylococcus aureus*: ATCC 25923) bacterial strains were used. Tryptic Soy Broth (Difco, USA) and Plate Count Agar (Difco, USA) were used. Solutions of working buffers (0.3 mM KH_2_PO_4_) were prepared from buffer stock solutions (0.25 M KH_2_PO_4_) by diluting 1 ± 0.1 mL with 800 mL of deionized water (DI).

### 2.2 Fabrication of Reticulated Open-Cell Mullite Foams

In this study, the silica powder derived from RH designated as nSiO_2_ was mixed with commercial alumina (Al_2_O_3_) by using the same method for producing the starting powders of the M2 formula (the fixed ratio of Al_2_O_3_:SiO_2_ from rice husk (RH) = 3:2 by weight) as reported in the previous study.^39,40^ Briefly, RH was biologically pretreated for a week and subsequently washed with water and DI water, dried at 80 °C and calcined at 650 °C to produce nSiO_2_. To prepare the aqueous mullite slip, Al_2_O_3_ and nSiO_2_ powders were mixed with 0.5 wt % poly­(vinyl alcohol) (PVA) as a binder. The mixture was milled with zirconia balls at 200 rpm for 144 h and dried at 80 °C to obtain the starting powder for slip preparation in the next step.

Reticulated open-cell mullite foams were fabricated by the replica technique, as explained in the previous study.^26,40^ 45 ppi polyurethane foams, used as the macropore template, were cut into approximate dimensions of 25 × 25 × 25 mm, coated with a slurry of the starting powder, dried, and sintered at a maximum temperature of 1500 °C for 4 h. Hereinafter, the sintered foams were coded as ROM, which was the designation used in the previous study^26^ using the differences in (i) the starting powders of the M4 formula (a fixed ratio of Al_2_O_3_:SiO_2_ derived from rice husk (RH) = 3:2 by weight, with 1 wt % of activated carbon (AC))^39,40^ and (ii) the cut foam size. Finally, the sintered foams were pretreated with a 6 M HCl solution before undergoing the dip-coating process to promote possibilities of the surface adhesion between the sintered foams and the GO layers. nSiO_2_ was used to replace commercial SiO_2_ powder, thereby recycling solid waste from the rice milling process. In addition, the use of nSiO_2_ in the fabrication of ROM foams has been reported to lower the sintering temperature and enhance mullite phase formation, resulting in improved compressive strength.^40^

### 2.3 Synthesis of GO Powders

GO powders used in this study were synthesized via the modified Hummers’ method, using commercially available pencil leads as the graphite source, as reported in our previous studies.^24,26,41^ Specifically, 3.0 g of the prepared graphite powder was added to an acidic solution of H_2_SO_4_ and H_3_PO_4_. KMnO_4_ used as the oxidizing agent, was then added to the mixture. The oxidation reaction was further carried out at 50 °C under continuous stirring for 36 h. Ice cubes made from DI (deionized) water and 30% H_2_O_2_ were then added to prevent further oxidation. The resulting solution was sonicated for 10 min and subsequently washed with 10% HCl and DI water until a neutral pH was reached, using the centrifugation method to wash the solution. Finally, the wet GO paste was separated from the solution and dried at 60 °C in an electric oven overnight.

### 2.4 Coating Processes

Multilayers of GO were coated on the foam surfaces by using the dip-coating method, as reported in a previous study.^26^ The coating method is simple and cost effective. The synthesized GO powder was mixed with deionized water to prepare a GO solution (0.5 g/L). Each foam was dip-coated in the prepared GO solution for 5 min, with care taken to obtain homogeneous GO layers adhering to the foam surfaces. The coated foams were then dried at 60 °C to complete the initial coating layer. The coating process was repeated for 3, 5, and 10 cycles. The coated foams were designated as 3-, 5-, and 10-GO/ROM for the GO-coated foams.

### 2.5 Antibacterial Activity Tests

The samples for testing antibacterials were divided into 2 types: powders and foams. The antibacterial activities of GO, uncoated, and GO-coated foams were tested based on a standard test of ASTM (E2149: Standard Test Method for Determining the Antimicrobial Activity of Antimicrobial Agents Under Dynamic Conditions).^6,8,42–44^ This standard method is one of the quantitative test methods used to evaluate nonleaching treated samples and microbial growth under dynamic contact conditions. In this study, the test method was modified for testing the antibacterial activity of the fabricated foams (1 piece of a foam sample/50 mL of bacterial inoculum), while the powder samples were tested according to the standard method (1.0 g of each powder sample/50 mL of bacterial inoculum). As shown in Figure 1, the tests were performed against *Escherichia coli* and *Staphylococcus aureus*. Uncoated foams, GO-coated foams, and inoculum without samples were designated as untreated, treated, and blank samples, respectively. Three replicates of the same samples were used and tested in this study. All samples were transferred to 250 mL glass bottles, capped loosely, sterilized in an autoclave (HVE-50, Hirayama, Japan) heated at 121 °C for 15 min, and finally dried in an electric oven at 80 °C overnight before running the tests. Bacterial inoculum in working buffer solutions was prepared and adjusted to approximately 1.5–3.0 × 10^5^ CFU/mL. 50 mL of the solution was added to the prepared bottle of samples and shaken using a shaker (GFL3005, GFL, Germany) at a speed of 200 rpm at 25 ± 2 °C for 24 h. After the specified time, an aliquot of the buffer from the bottle was pipetted into the sterile Pretri dish and poured with the plate count agar and incubated in an incubator (BD400, Binder, Germany) operated at 35 °C for 48 h. The number of surviving cells was counted, and the average of the duplicates was calculated as CFU/mL by multiplying by the dilution factor. Antibacterial activities were expressed as % reduction and Log10 bacteria reduction, as shown in Eqs 1 and 2), respectively.^43^

1 Reduction(%)=[(C−A)/C)]×100

2 $${\mathrm{Log}}_{10}\mathrm{bacterial}\;reduction=\mathrm{Log}\left(C\right)\;-\;\mathrm{Log}\left(A\right)$$

**Figure 1:**
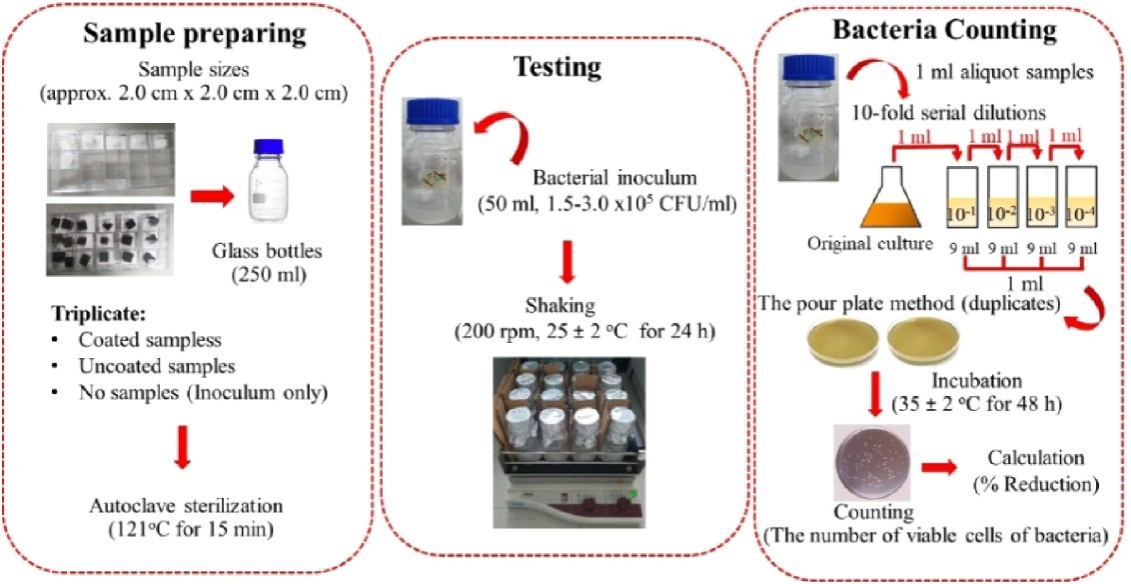
Experimental procedures for testing antibacterial activities of samples followed the test method of ASTM E2149, modified for testing the foams fabricated in this study.

where A is the CFU per milliliter for the flask containing the treated substrate after the specified contact time, and C is the CFU per milliliter for the flask containing the untreated substrate only after the specified contact time.

### 2.6 Characterization

The crystal structures of the synthesized GO were analyzed using an X-ray diffractometer (XRD, PANalytical X’Pert PRO, Netherlands), equipped with a Cu–Kα radiation source (40 kV, 30 mA). Diffraction patterns were recorded over the 2θ range of 5–70° at a scan speed of 2.4°/min. The chemical functionalities were examined using a dispersive Raman spectrometer (Senterra, Bruker Optics, Germany) with a 532 nm excitation laser operating at 20 mW. The Raman spectrum was collected with a TE-cooled CCD detector. The chemical states of the sample were characterized by X-ray photoelectron spectroscopy (XPS, AXIS Supra, Kratos Analytical Ltd., UK) using monochromatized Al–Kα radiation (1486.6 eV) at 15 keV and 15 mA. The chamber pressure was maintained below 10^–7^ Pa during the measurement. A wide-scan spectrum was collected with a pass energy of 160 and 1.0 eV resolution, while high-resolution spectra were collected with a pass energy of 20 and 0.1 eV resolution. Binding energies were calibrated to the C 1s peak at 285 eV. Data analysis and peak deconvolution were performed using ESCApe software (version 1.2.7501.7302).

Morphologies of GO powders and the foams were investigated by field emission scanning electron microscopes (FE-SEMs, JSM7800F Prime, JEOL, Japan, and SU5000, Hitachi, Japan). An X-ray Computed Tomography system (X-ray CT, Phoenix v|tome|x M 240, Waygate Technologies, Germany) was used to investigate the external and internal structures of the ROM and all GO/ROM samples. Typically, the porous characteristics of the characterized samples were primarily analyzed to investigate the GO-coated layers. The sample was placed on a 360° rotational stage, and a 3D image was captured by using a 70 kV accelerating voltage and a 220 μA current. Each X-ray CT analysis generated 2300 pictures, with each image projection requiring 334 ms. These 2D images were transformed into 3D tomographic images consisting of voxels with an edge length of 15 μm.^45^ For the porosity analysis, VGStudio MAX software (Volume Graphics, Heidelberg, Germany) was utilized for the computation of all parameters.

## 3 Results and Discussion

Figure S1a-d shows the characterization results for determining the structural properties of the synthesized GO. The resulting details are reported and discussed in the Supporting Information.

### 3.1 Characteristics of ROM and GO/ROM Foams

Figure 2 shows a digital photograph of the ROM foams before and after coating with the GO solution. It shows that the surface change of the foam, from shiny white to black, can be used as an indicator to confirm the successful GO coating. After 10 coating cycles, the foam coated with the GO solution was completely covered with GO, as evidenced by the fully black appearance of the 10-GO/ROM sample.

**Figure 2:**
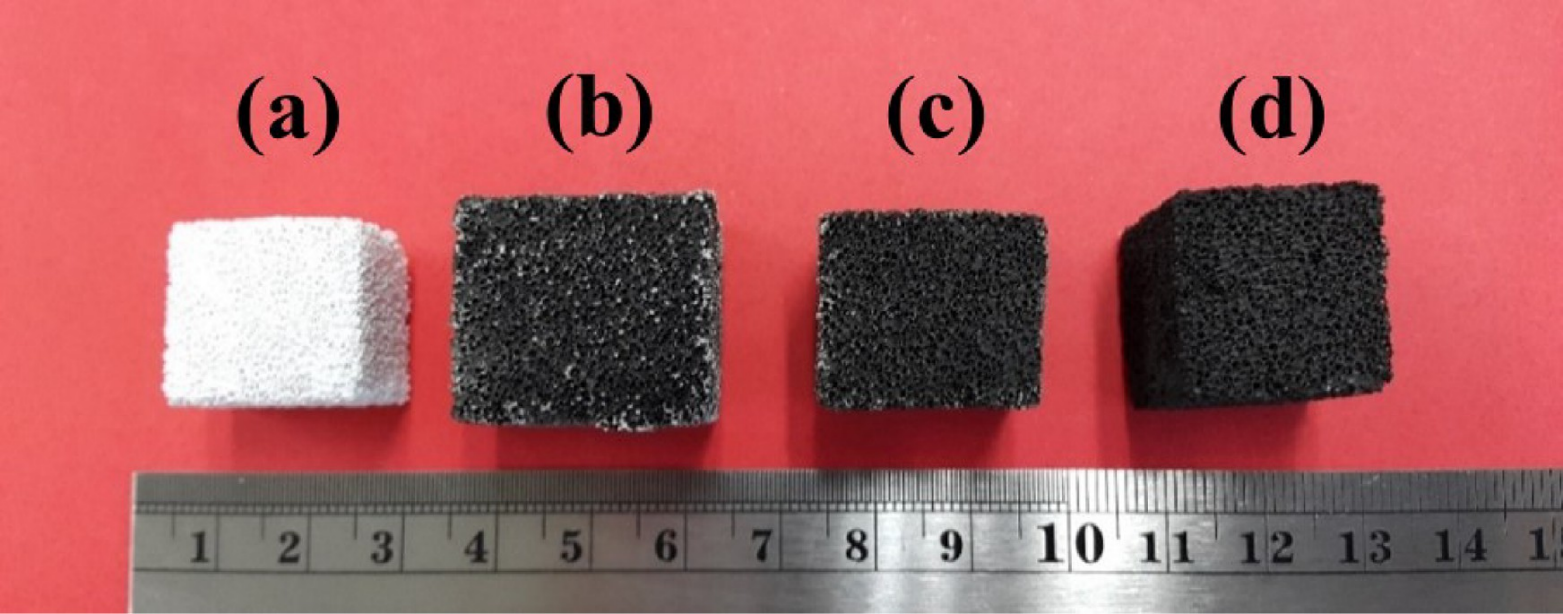
A digital photograph revealing the external characteristics of samples: (a) ROM, (b) 3-GO/ROM, (c) 5-GO/ROM, and (d) 10-GO/ROM.

### 3.2 External and Internal Microstructures

#### 3.2.1 SEM Investigations

Figure 3 compares the microstructure of the uncoated foam to those of the GO-coated foams observed by the FE-SEM investigations. Based on the observation, the uncoated foam exhibited a microporous structure of interconnected cellular architecture with minimal pore blockage (Figure 3a). However, the foam was able to maintain its original pore shape and struts even after the high-temperature sintering process (1500 °C for 4 h). The skeleton was entirely coated with uniformly distributed mullite ceramic particles, and small voids were observed within the ceramic matrix. Figure 3b-d presents the ROM foams coated with or covered by the GO layers after different coating cycles. The surfaces of the foams became rougher as the number of coating cycles increased. The wrinkled GO sheets were clearly seen after 3 cycles. By the 10th cycle of GO coating, the foam surface was almost completely covered with the GO layers, consistent with the visual observations in the photographs.

**Figure 3:**
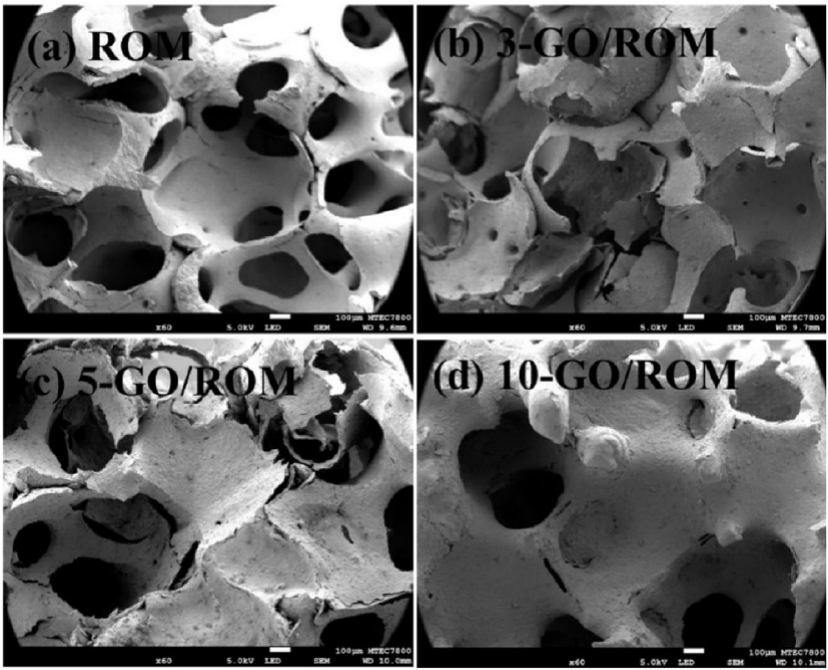
SEM images of (a) ROM, (b) 3-GO/ROM, (c) 5-GO/ROM, and (d) 10-GO/ROM.

At higher magnification of the microstructural observation, SEM images shown in Figure 4a reveal a rough and cracked microstructure on the surface of the ROM foam. After the GO coating, the mechanical flexibility of individual GO nanosheets, combined with their strong affinity for the ROM foam surface, facilitated the formation of a continuous layer of GO sheets, as shown in Figure 4b. However, the ceramic grains were still visible after three coating cycles. Figure 4c displays a wrinkled structure, indicating the presence of GO on the ROM surface. The GO nanosheets adhered well between layers, likely due to the self-assembly forces of van der Waals forces, hydrogen bonding, and π-π stacking.^46^ After 10 cycles of GO coating, the foam was fully covered with GO sheets (Figure 4d). Increasing the number of coating cycles resulted in a greater amount of GO on the foam, as confirmed by the increased weight of the foam with each additional cycle.

**Figure 4:**
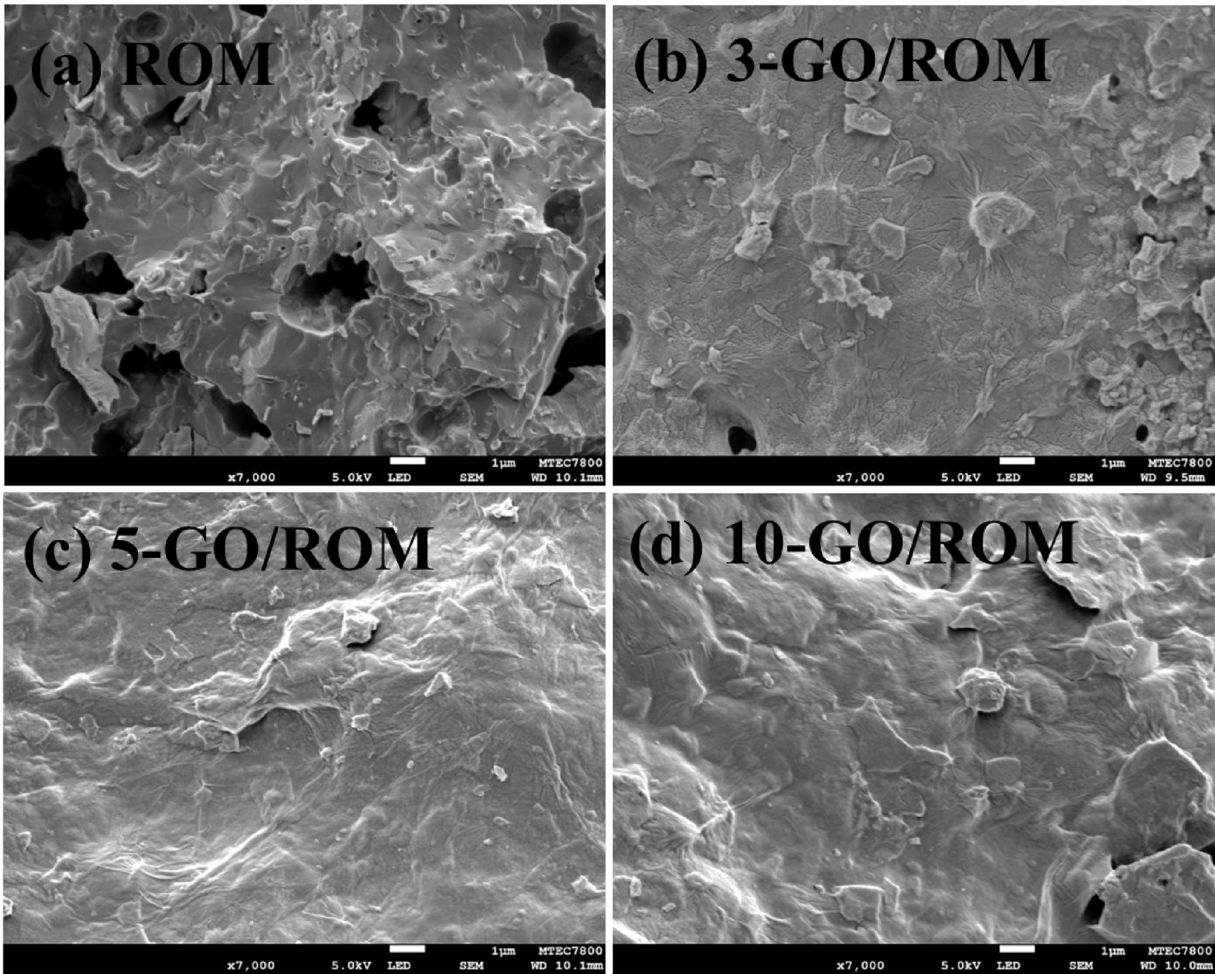
SEM images revealing the GO layers covering the ROM surfaces: (a) ROM, (b) 3-GO/ROM, (c) 5-GO/ROM, and (d) 10-GO/ROM.

#### 3.2.2 X-ray CT Analyzed Results

To investigate the distribution of GO on the morphological properties of the ROM foams, we conducted X-ray CT analysis. The external surface structures of the uncoated ROM foams and the GO-coated ROM foams are presented in Figure 5. The initial pore networks originating from the polyurethane template used in the fabrication process were well preserved in the sintered ROM foam, as shown in Figure 5a. This result is consistent with observations from SEM investigations, confirming that the ROM foam effectively replicated the original pore morphology of the template. Moreover, Figure 5b-d display the ROM foams coated with GO at varying thicknesses, revealing that the open-pore structure was maintained, which could facilitate the effective penetration and distribution of the GO solution within the foam frameworks.

**Figure 5:**
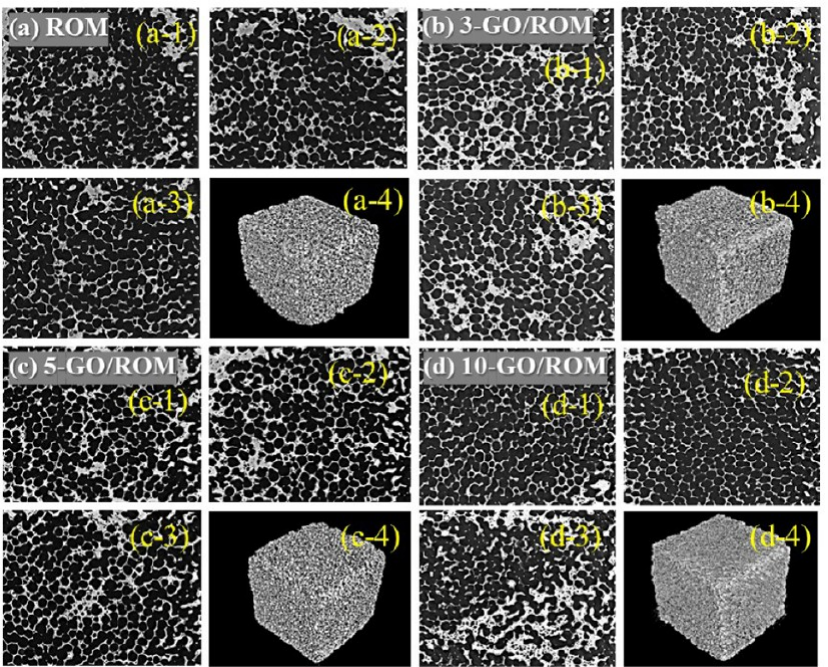
X-ray CT images showing X-sections of the scanned samples (−1, −2, −3) and overall images of the samples scanned at external surfaces (−4) of each sample: (a) ROM, (b) 3- GO/ROM, (c) 5-GO/ROM, and (d) 10-GO/ROM, respectively.

Figure 6 presents cross-sectional images of the uncoated ROM foam compared with the GO-coated ROM foams. Variations in the grayscale color were considered and could represent differences in the relative density of materials, with high-density ceramic regions of the ROM foam appearing white and air regions appearing dark. Typical characteristics of the ROM foam, including porosity (macropores) and triangular hollows within struts, are clearly seen in Figure 6a. These features commonly observed in open-cell ceramic foams. After the GO coating (Figure 6b-d), thin, dark gray layers formed on the cell walls, indicating the successful deposition of GO films on the foam surface. It was found that this GO coating reduced the pore sizes compared to the uncoated ROM foam. However, small gaps visible beneath the GO layers suggested potential coating delamination, likely due to the surface roughness of the ROM foams occurring after the sintering process. The GO layer thicknesses on the foams were determined, as shown in Figure 6.

**Figure 6:**
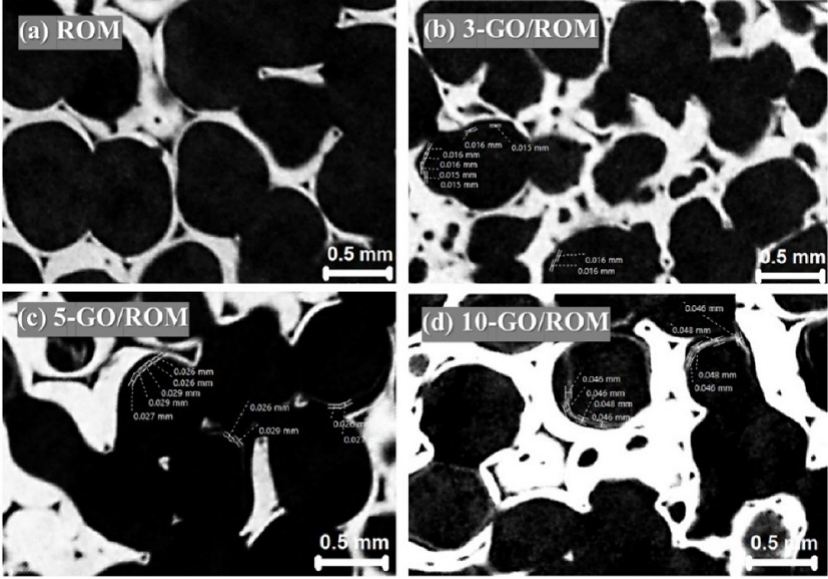
Micro-CT images showing the GO layer characteristics and the layer thickness measured for the coated foams, in comparison with the uncoated foam: (a) ROM, (b) 3-GO/ROM, (c) 5-GO/ROM, and (d) 10-GO/ROM.

Figure 7 shows the measurement of the gap distances between the layers and foam surfaces, as presented in the images. Table 1 summarizes the values of macropore sizes, macropore volumes (%), layer thicknesses, and gap distances measured by using the micro-3D scan images. The average thicknesses increased from 16 to 47 μm as the number of GO coating cycles increased from 3 to 10 cycles. The ROM foams exhibited an average macropore size of 0.78 mm and a macropore volume of 0.32 mm^3^, corresponding to 81.97% of the total volume. Upon GO coating, both the pore size and volume decreased, as observed in the 3-GO/ROM foam. Although the change in macropore characteristics between 3- and 5-GO coating cycles was minimal, a significant reduction in the macropore size and volume was found in the 10-GO/ROM foam. This finding confirms that increasing the GO layer thickness effectively reduces macropore size and volume, consistent with the cross-sectional CT images presented in Figures 6 and 7. Consequently, increased GO content might benefit the antibacterial properties of the coated foams.

**Figure 7:**
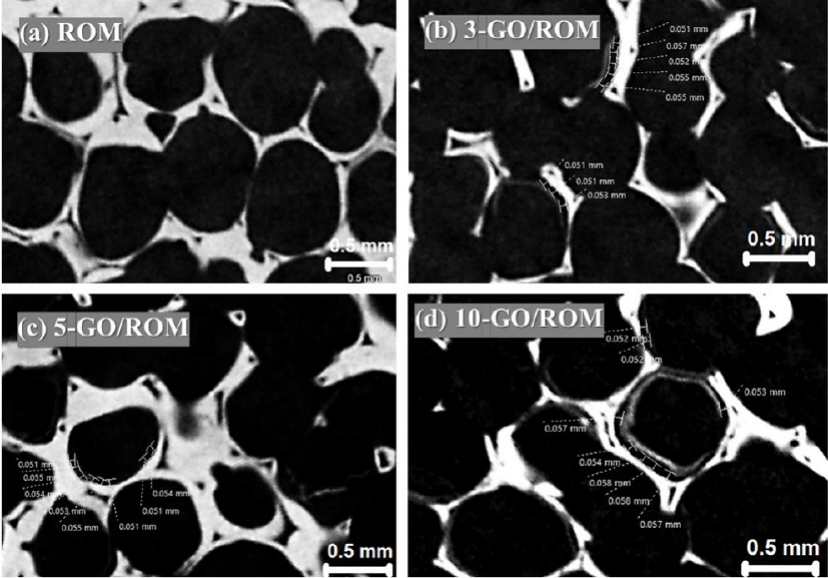
Micro-CT images showing the gap distances measured between the foam surfaces and the coating layers of the coated foams, in comparison with the uncoated foam: (a) ROM, (b) 3-GO/ROM, (c) 5-GO/ROM, and (d)­10-GO/ROM.

**Table 1:** Pore Characteristics, GO Thicknesses, and the Gap Distances between the Foam Surfaces and the GO Layers, Calculated from Micro-3D Scanned Images

	Macropores				
Samples	Sizes^a^ (mm)	Volumes^a^ (mm^3^)	Macropore volumes (%)^a^	Thicknesses of GO layers (mm)^a^	Gap distances between the ROM surfaces-GO layers (mm)^a^
**ROM**	0.780 ± 0.219	0.315 ± 0.126	81.97	-	-
**3-GO/ROM**	0.719 ± 0.265	0.294 ± 0.137	81.50	0.016 ± 0.001	0.053 ± 0.002
**5-GO/ROM**	0.783 ± 0.240	0.332 ± 0.148	81.72	0.027 ± 0.001	0.053 ± 0.002
**10-GO/ROM**	0.588 ± 0.188	0.142 ± 0.121	81.57	0.047 ± 0.001	0.055 ± 0.003

a Average values ± SD.

### 3.3 Antibacterial Activity Evaluation

Antibacterial activities of samples against Gram-negative bacteria, *E. coli* (ATCC 25922), and Gram-positive bacteria, *S. aureus* (ATCC 25923), were evaluated following the ASTM E2149 standard. Both powder and foam samples were tested to assess the adhesion effects on different surfaces. Figure 8a compares the viable *E. coli* count before and after 24 h of incubation. The bacterial reduction was influenced by the surface morphology. In powder samples, the number of viable bacteria significantly decreased in the presence of the synthesized GO powder and the ROM foam (ground and used in powder form, ROM, Lot no.: 1500SC4), achieving more than 99% reduction compared to the control. Notably, GO exhibited stronger antibacterial activity than powdered ROM, consistent with the absence of *E. coli* colonies in the Petri dish images (Figure 8b-d). The powdered ROM exhibited a higher viable bacterial count than the control sample, likely due to residual bacterial growth after cell adhesion, which led to an overall increase of 4.10%. In contrast, GO-coated foams demonstrated significant antibacterial effects, with bacterial reduction rates of up to 99.89% for 3-GO/ROM and 5-GO/ROM, and over 99.99% for 10-GO/ROM. Strong evidence from Figure 8e-h indicates a correlation between GO layer thickness and antibacterial efficacy, as thicker GO coatings resulted in fewer bacterial colonies. The absence of bacterial colonies on the 10-GO/ROM plate confirmed its superior antibacterial performance.

**Figure 8:**
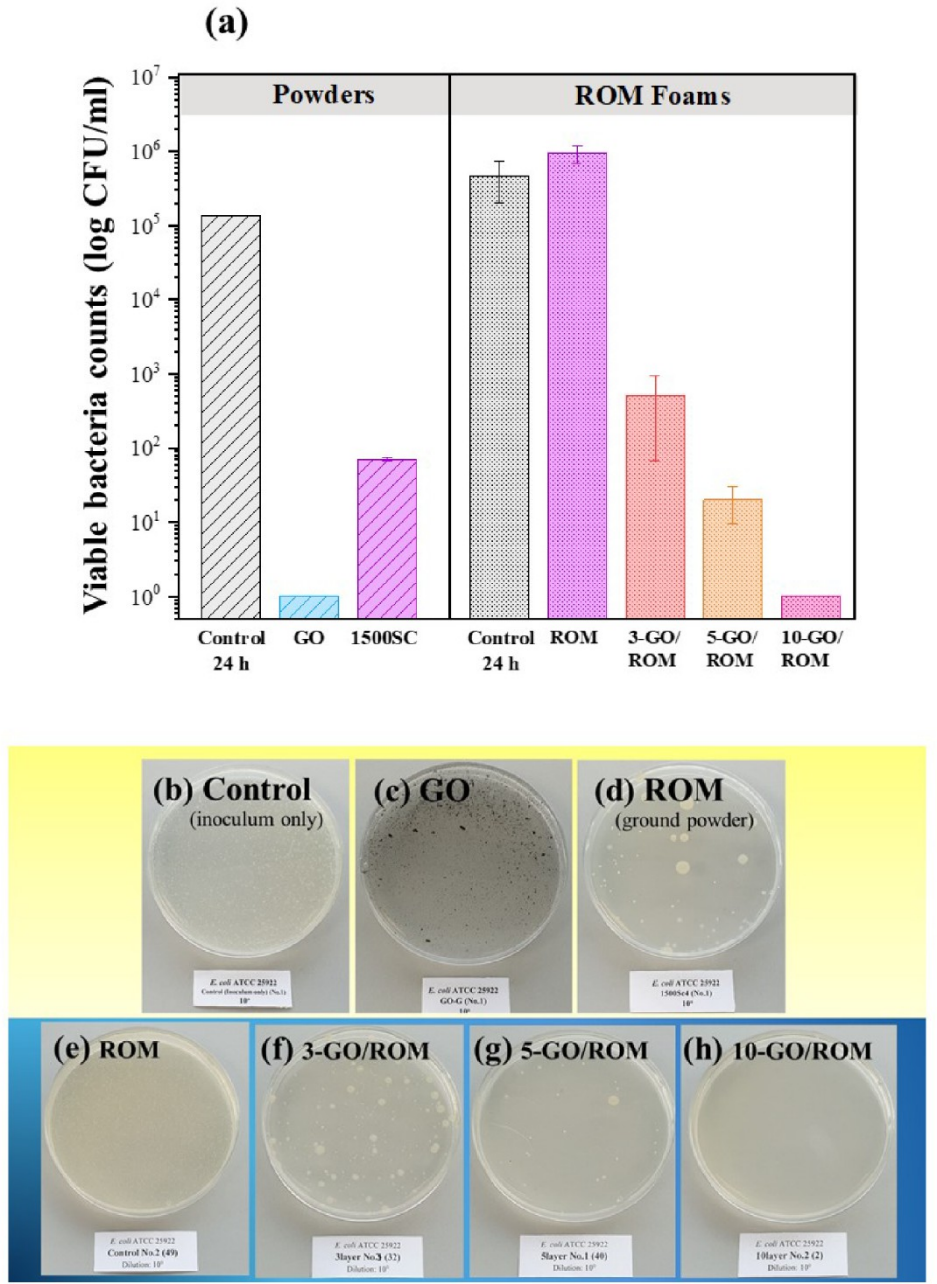
(a) Viable cell counts of *Escherichia coli* ATCC 25922 after a contact time of 24 h, in comparison with the antibacterial properties of powder and foam samples and (b)-(h) corresponding digital photographs of agar culture plates after cultivating *E. coli* colonies. The agar culture plate representing the control samples (inoculum only), tested with the ROM and GO-coated ROM samples, was not shown in the figure because the ROM samples were used as control samples.

The antibacterial activity against *Staphylococcus aureus* (ATCC 25923) is revealed in Figure 9a. The results indicated that the GO powder exhibited a strong antibacterial property, inhibiting Gram-positive bacteria by 99.5%, whereas the ROM powder achieved only a 17.0% reduction. When considering all the foam samples, the uncoated foam demonstrated a 78.12% reduction in *S. aureus*. However, after the GO coating, antibacterial efficacy significantly improved, with the 10-GO/ROM foam achieving a 98.87% reduction, as strongly evidenced by the substantial decrease in bacterial colonies, as shown in Figure 9b-h.

**Figure 9:**
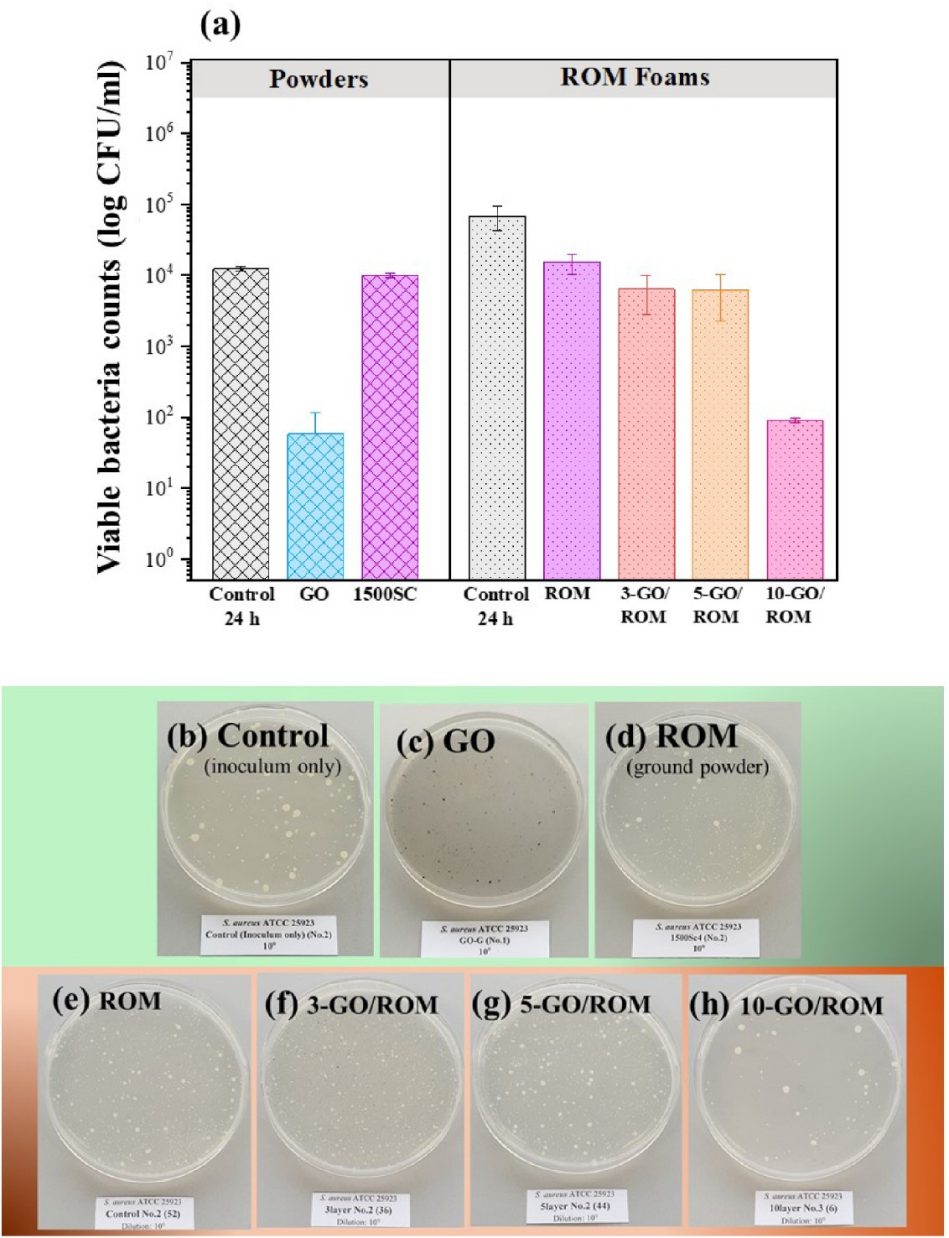
(a) Viable cell counts of *Staphylococcus aureus* ATCC 25923 after 24 h of contact time, in comparison with the antibacterial properties of powder and foam samples and (b)-(h) corresponding digital photographs of agar culture plates after cultivating *S. aureus* colonies. The agar culture plate representing the control samples (inoculum only), tested with the ROM and GO-coated ROM samples, was not shown in the figure because the ROM samples were used as control samples.

Interestingly, the inhibitory effect of the GO-coated foams is more prominent against *E. coli* than against *S. aureus*. The increase in viable *E. coli* in the ROM foam after incubation may be attributed to the reticulated structure’s ability to absorb liquid media, creating a nutrient-rich environment that promotes bacterial growth. This phenomenon is consistent with findings by Dutto et al., which reported that porous ceramic scaffolds could support *E. coli* proliferation, a property beneficial for gas-sensing applications. Their study demonstrated that a specific *E. coli* strain exhibited exponential growth, reaching 10^6^–10^7^ mL^–1^ within the first 5 h of the experiment.^47^

### 3.4 Mechanism of Antibacterial Activity

To find out the antibacterial mechanism of the GO-coated ROM foams, the interactions between the ROM foams and GO/ROM with *E. coli* and *S. aureus* were further investigated by SEM analysis. Figure 10a,c shows viable *E. coli* cells adhered to the ROM surface, exhibiting elongated morphology with lengths of approximately 1.0–1.5 μm. In contrast, the cells on the 10-GO/ROM foam surface display irregular shapes and cell depression (Figure 10b), indicating a loss of cellular integrity. Figure 10d reveals *E. coli* cells encapsulated by GO layers coated on the foam. These morphological disruptions are consistent with previous findings by Liu et al.^48^

**Figure 10:**
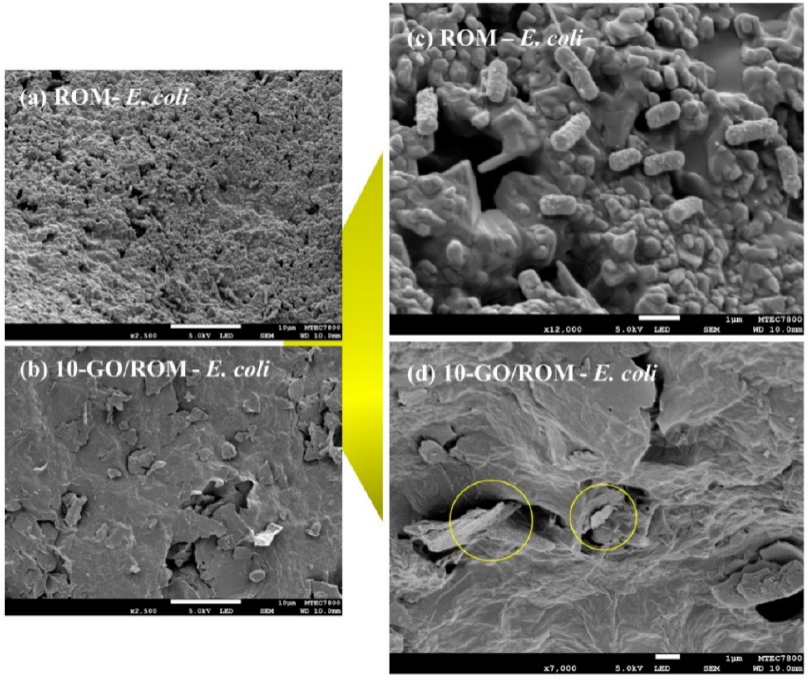
(a)-(d) SEM images of typical samples after the antibacterial tests of *E. coli*. The images (c) and (d) were taken at higher magnification than those of images (a) and (b).

Similarly, Figure 11a,c depicts typical *S. aureus* cells on the ROM foam with a smooth surface and spherical shape (0.8–1.0 μm), representative of live cells. Notably, a single cell was rarely observed, as they predominantly appear in binary forms or clusters, indicating active proliferation. Figure 11b,d present the foam surface after incubation with 10-GO/ROM, and few residual bacterial cells remain on the foam surface, suggesting poor bacterial adhesion.

**Figure 11:**
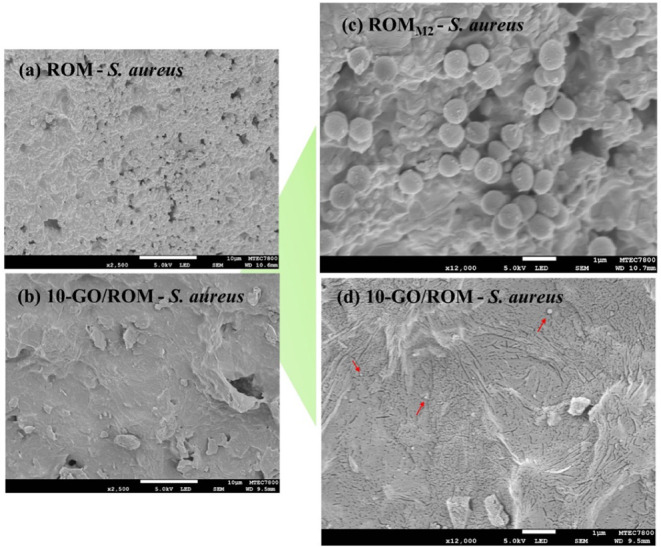
SEM images of typical samples after the antibacterial tests of *S. aureus* (a)-(d). The images (c) and (d) were taken at higher magnification than those of the images (a) and (b).

The bactericidal mechanism of the GO/ROM foams is probably attributed to the interaction between GO and bacterial cells. GO contains oxygenated functional groups such as hydroxyl, carbonyl, and epoxy, which can interact with lipids and proteins in the bacterial cell wall or membrane through hydrogen bonding, π–π stacking, or electrostatic interactions. These interactions may lead to lipid extraction, protein denaturation, or the generation of reactive oxygen species, resulting in cell membrane disruption and microbial inactivation.^49,50^ Additionally, bacterial cells can be physically trapped within the GO layers in the porous structure of the GO/ROM, as illustrated in Figure 10d. This trapping effect likely limits nutrient diffusion, thereby inhibiting bacterial proliferation. A similar mechanism was reported in the work of Kanchanapally et al., where a three-dimensional porous GO membrane with approximately 300 nm pores demonstrated bactericidal activity against *S. aureus* via mechanical wrapping and subsequent membrane disruption.^51^

Interestingly, the antibacterial efficacies of GO/ROM foams were significantly greater against *E. coli* than *S. aureus*, as shown in Figures 8a and 9a. This difference may be explained by structural differences between the two bacteria. *E. coli* possesses a thin peptidoglycan layer with an outer membrane, making it more susceptible to GO disruption, whereas *S. aureus* has a much thicker peptidoglycan layer, measuring several tens of nanometers, which provides greater resistance.^49^ Furthermore, the larger cell size of *E. coli* may enhance its contact area with the GO-coated foams, facilitating more extensive cell disruption.

## 4 Conclusions

In this study, 3D interconnected porous mullite foams (reticulated open-cell mullite foams) were fabricated and coated with synthesized graphene oxide (GO) solutions via a dipping technique, repeated for 3, 5, and 10 cycles, to study the influence of GO layer thickness on antibacterial performance. Due to the external color of the mullite foams changing from white to black, it was confirmed that the coating technique was successful. X-ray computed tomography and SEM analyses demonstrated uniform GO coverage within the open-cell network, with increasing cycle numbers resulting in thicker GO coatings and reduced macropore size. Antibacterial testing against Gram-negative *E. coli* and Gram-positive *S. aureus* revealed a clear dose–response effect. By increasing GO layers from 3 to 10 cycles, the fabricated foams achieved up to 99.99% reduction in viable *E. coli* and 98.87% reduction in *S. aureus* within 24 h of incubation. On the other hand, uncoated mullite foams exhibited negligible or even increased bacterial counts, likely due to nutrient entrapment within their reticulated structure. The bactericidal mechanism was investigated through SEM observation, suggesting that the oxygenated functional groups of GO play a role in disrupting bacterial cell walls or cell membranes, while physical trapping within the GO-coated layer limits nutrient access, resulting in cell inactivation. These findings highlight GO-coated mullite foams as promising antibacterial materials.

## Supplementary Material



## Notes

Structural properties of the synthesized GO by XRD, Raman, and XPS analysis (PDF)
