# Supplementary material for: Graphene Oxide (GO) Coating on Reticulated Open-Cell Mullite (ROM) Foams for Enhancing Antibacterial Activity
Source: ACS Omega. 2025 Nov 4;10(45):53969–79. doi: 10.1021/acsomega.5c04259 (PMC12631316; doi:10.1021/acsomega.5c04259)
Supplement: Supplementary file 1 [file ao5c04259_si_001.pdf]

Supporting Information

Graphene Oxide (GO) Coating on Reticulated Open-Cell Mullite (ROM) Foams for Enhancing Antibacterial Activity

Wadwan Singhapong, Angkhana Jaroenworaluck*,

Watchara Chokevivat*, Pongthorn Suksanong

National Metal and Materials Technology Center (MTEC),

National Science and Technology Development Agency (NSTDA),

111 Thailand Science Park, Phahonyothin Road, Khlong Nueng, Khlong Luang,

Pathum Thani, 12120, Thailand

*Corresponding authors:

1. Angkhana Jaroenworaluck (angkhanj@mtec.or.th)

2. Watchara Chokevivat (watcharc@mtec.or.th)

Tel.: +66 2 546 6500

Structural Properties of Graphene Oxide (GO)

The chemical properties of graphene oxide (GO) synthesized from pencil leads as the graphite source, were characterized by XRD, Raman spectroscopy, and XPS, as presented in Figure S1a-d. Figure S1a shows a diffraction peak at 8.80º 2θ corresponding to the (001) crystal plane of GO with an interlayer spacing of 1.0 nm. A weak and broad (002) graphite diffraction peak is observed at ~20º 2θ. Figure S1b presents the Raman spectrum where a prominent G band at 1596 cm^{-1} corresponds to the in-plane stretching vibration of sp^2 carbon. A weaker D band at 1347 cm^{-1} indicates the presence of structural defects arising from oxygenated functional groups introduced during the oxidation process.[1] Figure S1c displays the wide scan XPS spectrum of GO showing C and O as the major elements. Notably, a minor S 2p peak is also detected, attributed to residual H_2SO_4 impurities remaining. Small Si 2p and Al 2p peaks are observed, likely originating from the clay binder in graphite pencil leads.[2] These impurities may enhance interfacial adhesion between GO layers by forming crosslinks, thereby improving the structural stability of GO.[3,4] The deconvoluted C 1s spectrum (Figure S1d) further confirms the presence of various oxygen functional groups, including aromatic rings (C-C/C=C), epoxy and hydroxyl (C-O), carbonyl (C=O), and carboxyl groups (O=C-O). It verifies the successful oxidation of graphite to GO.

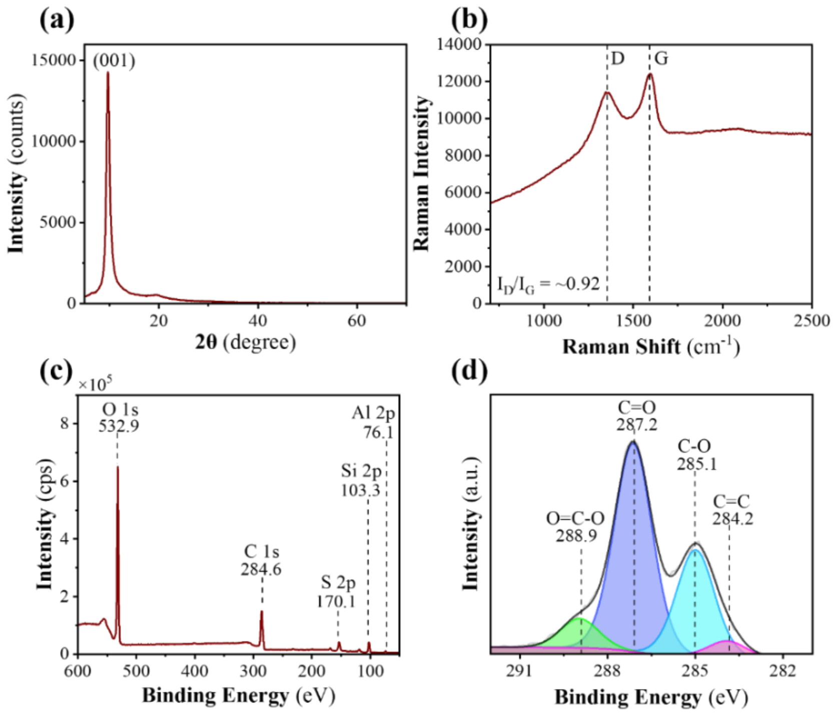

Figure S1. Properties of the synthesized GO: (a) the XRD profile, (b) the Raman spectrum, (c) the XPS wide scan spectrum and (d) the deconvoluted C 1s spectra.

References

(1) Marcano, D. C.; Kosynkin, D. V.; Berlin, J. M.; Sinitskii, A.; Sun, Z.; Slesarev, A.; Alemany, L. B.; Lu, W.; Tour, J. M. Improved synthesis of graphene oxide. *ACS Nano* **2010**, *4*, 4806-4814.

(2) Bernalte, E.; Foster, C. W.; Brownson, D. A. C.; Mosna, M.; Smith, G. C.; Banks, C. E. Pencil it in: Exploring the feasibility of hand-drawn pencil electrochemical sensors and their direct comparison to screen-printed electrodes. *Biosensors* **2016**, *6*, 45.

(3) Yeh, C.-N.; Raidongia, K.; Shao, J.; Yang, Q.-H.; Huang, J. On the origin of the stability of graphene oxide membranes in water. *Nature Chemistry* **2015**, *7*, 166-170.

(4) Singhapong, W.; Jaroenworaluck, A.; Manpetch, P. Novel and reusable graphene oxide-coated reticulated open-cell mullite foams for methylene blue dye adsorption. *ACS Omega* **2024**, *9*, 5541-5547.
